# Cannabis, alcohol and fatal road accidents

**DOI:** 10.1371/journal.pone.0187320

**Published:** 2017-11-08

**Authors:** Jean-Louis Martin, Blandine Gadegbeku, Dan Wu, Vivian Viallon, Bernard Laumon

**Affiliations:** Univ Lyon, Université Claude Bernard Lyon 1, Ifsttar, UMRESTTE, UMR_T 9405, Bron, France; Waseda University, JAPAN

## Abstract

**Introduction:**

This research aims to estimate the relative risks of responsibility for a fatal accident linked to driving under the influence of cannabis or alcohol, the prevalence of these influences among drivers and the corresponding attributable risk ratios. A secondary goal is to estimate the same items for three other groups of illicit drugs (amphetamines, cocaine and opiates), and to compare the results to a similar study carried out in France between 2001 and 2003.

**Methodology:**

Police procedures for fatal accidents in Metropolitan France during 2011 were analyzed and 300 characteristics encoded to provide a database of 4,059 drivers. Information on alcohol and four groups of illicit drugs derived from tests for positivity and potential confirmation through blood analysis. The study compares drivers responsible for causing the accident, that is to say having directly contributed to its occurrence, to drivers involved in an accident for which they were not responsible, and who can be assimilated to drivers in general.

**Results:**

The proportion of persons driving under the influence of alcohol is estimated at 2.1% (95% CI: 1.4–2.8) and under the influence of cannabis at 3.4% (2.9%-3.9%). Drivers under the influence of alcohol are 17.8 times (12.1–26.1) more likely to be responsible for a fatal accident, and the proportion of fatal accidents which would be prevented if no drivers ever exceeded the legal limit for alcohol is estimated at 27.7% (26.0%-29.4%). Drivers under the influence of cannabis multiply their risk of being responsible for causing a fatal accident by 1.65 (1.16–2.34), and the proportion of fatal accidents which would be prevented if no drivers ever drove under the influence of cannabis is estimated at 4.2% (3.7%-4.8%). An increased risk linked to opiate use has also been found to be significant, but with low prevalence, requiring caution in interpreting this finding. Other groups of narcotics have even lower prevalence, and the associated extra risks cannot be assessed.

**Conclusion:**

Almost a decade separates the present study from a similar one previously conducted in France, and there have been numerous developments in the intervening years. Even so, the prevalence of drivers responsible for causing fatal accidents under the influence of alcohol or narcotics has stayed remarkably stable, as have the proportion of fatal accidents which could in theory be prevented if no drivers ever exceeded the legal limits. The overall number of deaths from traffic accidents has dropped sharply during this period, and the number of victims attributable to alcohol and/or cannabis declined proportionally. Alcohol remains the main problem in France. It is just as important to note that one in two drivers considered to be under the influence of cannabis was also under the influence of alcohol. With risks cumulating between the two, it is particularly important to point out the danger of consuming them together.

## Introduction

Ever since the Grand Rapids study [1], all published research, whether experimental or observational, has shown a higher accident risk for drivers under the influence of alcohol [2–5]. This strongly increased risk can chiefly be explained by greatly reduced attentional and cognitive capacities, a delay in taking actions that could avoid an accident, and a higher degree of risk-taking [6], in particular, driving at high speed.

In recent years, there have also been numerous studies into driving under the influence of illicit drugs, notably cannabis. A number of experimental investigations have shown a decreased capacity of drivers under the influence of cannabis [7–9], in particular a decrease in attention, increased reaction time and reduced ability to control direction [10]. Individual variations are considerable, but there is an overall diminution in cognitive and motor functions related to driving. A further dose-dependent effect has been demonstrated in certain aspects of vehicle control, such as steering, keeping distance from the vehicle ahead, driving speed, reaction time and keeping on the right side of the road [11].

These experimental studies are indispensable for knowing how consumption of a given substance affects driving, and the intensity of its effect. Their main advantage is that they control both drivers’ actual consumption and driving conditions. On the other hand, for obvious ethical reasons, the doses to which the consumers are experimentally subjected are limited to reasonable levels, which in real life are often widely exceeded, for illegal drugs as well as alcohol. Moreover, in spite of constant technical progress in driving simulators, it is difficult for drivers to forget they are not on a real road, and that an error would not pose a danger as it would in real-life conditions. Driving on a test track is closer to reality, but still does not completely avoid the same criticism, given that courses are pre-established and drivers know they are being supervised. Finally, under controlled conditions, it is not certain that drivers adapt to their perceived capacities in the same way as in a real-life driving situation.

For all of these reasons, real-life observation studies are essential. Furthermore, they can estimate the prevalence of driving under the influence of alcohol or illegal drugs. Hitherto published research essentially falls into two categories: studies on responsibility, and case-control studies. In a recent study, Hartman differentiated the design of epidemiological studies in a review of the literature [12]. Ten case-control studies were examined, in which the control subjects were drivers not involved in any accident. Six of these case-control studies relied on self-report to determine use of cannabis. According to the authors, this would tend to minimize the odds ratio associated with driving under the influence when it comes to use of an illegal substance. For all of these case-control studies, the reliability of the results depends on the comparability of cases and controls. Studies based on determining responsibility, and assessing the increased risk of being responsible for an accident while under the influence, have the advantage of directly measuring the substances consumed in the two groups. They involve, however, an estimate of responsibility that requires knowledge of numerous elements of the circumstances of the accident. This was the method selected for the present study.

Meta-analyses have been published, based on the “best” papers, to produce reliable estimates of the risks linked to driving under the influence. Three recent reviews [13–15] in particular confirmed the order of magnitude of increased risk associated with driving under the influence of cannabis, estimated in France at 1.78 (95% CI: 1.40–2.25) [16].

Elvik’s review [15] focused on the influence of numerous legal and illegal drugs. The odds ratio (OR) connected to cannabis is estimated at 1.26 (95% CI: 0.88–1.81) when publication bias is taken into account using a methodology described by the authors. Another review of the literature [13] recalculated the crude risks for road users who had not consumed alcohol, so as to isolate the effect of cannabis in all the studies, which were selected according to quality standards specified in the article. In this way, the OR was re-estimated at 1.92 (95% CI: 1.35–2.73). Finally, the review by Li [14] estimated the crude OR at 2.66 (95% CI: 2.07–3.41), based on a selection of 9 studies that compared drivers involved or not involved in an accident. Five of the nine studies used self-reporting to estimate cannabis consumption.

The advantage of estimates derived from meta-analyses lies in their quantitative synthesis of numerous and sometimes contradictory results. One of the issues is however that the results are often weighted from crude relative measures, or from measures adjusted for very different factors.

In particular, it appears difficult for the risk linked to cannabis use not to take into account the consumption of alcohol with which it is often associated [17]. In France during 2002–2003, almost half of drivers involved in fatal crashes and found to be under the influence of cannabis were also under the influence of alcohol. As alcohol is associated with a higher OR, the risk associated with cannabis in the presence of alcohol is multiplied by this OR even in the absence of any positive interaction. It has further been shown in experimental conditions that drivers under the influence of alcohol tend to drive faster [18], which goes hand in hand with an overestimation of their own capacities [10], whereas drivers under the influence of cannabis tend to drive more cautiously [19,20]. Therefore, priority should be given to studies on the influence of using cannabis while driving that concomitantly assess the influence of alcohol.

## Objectives

The main objective of this research was to estimate the relative risks of responsibility for a fatal accident linked to driving under the influence of cannabis or alcohol, the prevalence of these influences when driving, and the attributable risk fractions to which they correspond.

Two secondary objectives were to estimate the same items for three other families of illegal narcotics (amphetamines. cocaine and opiates) and to compare the different results with those of a similar study conducted in France between 2001 and 2003 (the SAM study).

## Materials and methods

As part of the VOIESUR project (http://www.agence-nationale-recherche.fr/?Projet=ANR-11-VPTT-0007), a database was formed from thorough analysis and meticulous encoding of police procedures, digitized and centralized by an agency (TransPV) that supplies insurance companies with police records of traffic accidents. As the need arose, data collection services provided important elements that were missing, such as accident diagrams, photographs of the vehicles involved, and injury assessments.

**Fig 1:**
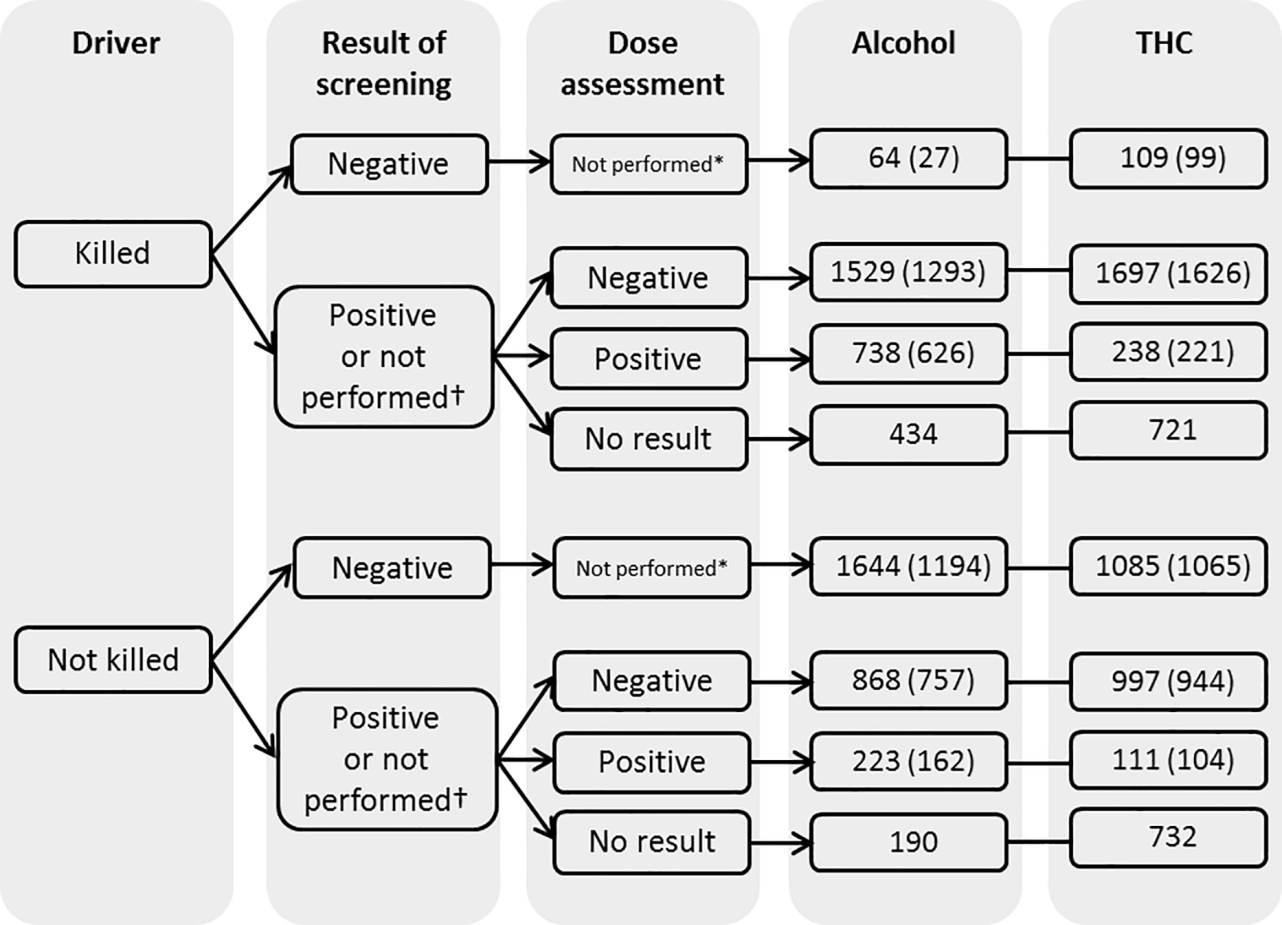
Screening and drug concentration measure process for drivers killed within 30 days after the crash or not. Numbers of drivers assessed positive, negative or with unknown status for alcohol and THC. * Dose assessment was not performed because the result of screening was negative. † Dose assessment was made in case of positive results of screening or when screening was not performed.

The collected data concerned all fatal accidents (with at least one people dead on the spot or within the 30 days following the road accident) occurring in Metropolitan France in the year 2011. The database therefore included 3,622 accidents described through more than 300 variables. Also encoded were the configuration and situation of accidents, human dysfunction, maneuvers of road users prior to the accident, collisions during the accident as well as any conflicts identified as playing a role in the occurrence of the accident. Conversely, information about driver health status was rarely reported by the police, nor possible confounding factors such as cell phone use or other driver distraction factors. With regard to narcotics, information was available on cannabis, opiates, amphetamines and cocaine. The method of data collection was indicated, and the drug concentration was measured, when possible, for all drivers killed as well as survivors found positive on detection tests. Fig 1 shows the numbers of drivers under influence or not (or unknown status) for alcohol and THC. The process is described separately for drivers killed within 30 days after the crash or alive in the same period, as the screening was most of the time impossible for the formers. Numbers of drivers with known alcohol and drug are indicated in parenthesis. For example, among the 738 killed drivers found positive for alcohol after blood analysis (BAC>0.5g/l), 626 have a known drug status. The final work sample includes 4059 drivers.

Estimating the degree of intoxication for cannabis and other narcotics is a trickier task than for alcohol. As far as cannabis is concerned, the effects vary rapidly with time. There is a rapid rise in the first fifteen minutes after consumption, followed by a fairly rapid decline [7]. The variety of substances and their means of consumption can also pose problems of measurement. However, the active ingredient behind most of the effects of cannabis that impair driving ability is THC. Metabolites such as THC-COOH are present and detectable for a significant time after consumption, but lack any proven psychoactive effects capable of impairing driving ability [21]. Cannabis intake is initially assessed either by urine or saliva test; confirmation and blood concentration are obtained through blood sampling.

Screening the urine or saliva of each driver yields either a ‘positive’ or ‘negative’ reading. If a result is positive, or urine/saliva testing is impossible, a blood sample is taken from the driver, which indicates the blood concentration. Only the result of the blood sample is deemed proof of positivity. In order to determine the narcotic status of the driver, the following rule is therefore applied:

- If a blood test was made and the result is known, then the narcotic status is established from this value. If it is above, or equal to, the legal limit, the driver is classified positive or, if not, negative.
- If the test has not been performed (impossible, refused, not carried out), the result was not known, and screening has proved negative, then the driver is classified negative for this substance.
- In all other cases, narcotic status is unknown.

The minimum regulatory for detection thresholds in urinary and salivary tests for the different families of narcotics are as follows:

- cannabis-related (9-THCCOOH), 50 ng/ml urine, 15 ng/ml saliva;
- amphetamine-related (amphetamine, methamphetamine, MDMA), 1000 ng/ml urine, 50 ng/ml saliva;
- cocaine-related (cocaine, benzoylecgonine), 300 ng/ml urine, 10 ng/ml saliva;
- opioids (morphine, 6 monoacetylmorphine), 300 ng/ml urine, 10 ng/ml saliva.

Any driver whose blood tests above or equal to the prescribed level for one or other of the classified narcotic substances is deemed positive for narcotics. Blood positivity levels are shown in the results tables. For cannabis, THC is screened.

To determine alcohol status, the driver must first submit to an alcohol screening test. Where the result is positive, or the test is refused, impossible or not carried out, the driver’s blood alcohol is measured, either by blood sampling (blood test) or breathalyzer (test of exhaled air). In the latter case, the value obtained (in mg/l of air) is multiplied by 2 in order to obtain an equivalence with the blood test (in g/l of blood).

### Determination of responsibility

The principle of a responsibility (or culpability) study [22,23] used here is to compare a group of drivers considered responsible for an accident, by having exhibited behavior (action or lack of action) which may have directly contributed to the accident’s occurrence, with a group of drivers involved in an accident for which they were not responsible.

The responsibility of a road user is not defined here in a legal sense. Rather, a person who contributes to or causes an accident is considered responsible, perhaps by an inappropriate maneuver (wrong-way driving, ignoring a traffic light, evident loss of control, *etc*.) or through failure (braking too late, forgetting to switch on headlamps, *etc*.). It is, of course, essential that the definition of responsibility is directly based on these behaviors, and not on their causes, which may be, for example, the driver’s inexperience or advanced age, use of a mobile phone while driving, or especially, driving under the influence of drugs and/or alcohol. Otherwise, the effects of these factors on the risk of responsibility would be greatly overestimated.

Responsibility was determined by a team of experts, drawing on all the facts at their disposal, including plans of the accident, and the comments of those involved and of the police. This gave a criterion of responsibility that was both reliable (in the “contribution” sense) and as objective as possible (based on facts).

### Composition of the two groups to be compared

Responsibility was ranked in 5 categories: 1—Totally responsible, 2 –Rather responsible, 3 –Shared responsibility, 4 –Rather not responsible, 5 –Not at all responsible. The responsible group included drivers whose responsibility was ranked 1, 2 or 3. This includes “rather responsible” and “shared responsibility” for the following reason: accidents often occur because of the conjunction of several factors, and removing one of those factors is usually enough to stop the accident occurring. Put another way, the accident would not have taken place if one or other of the drivers had not done something that leads an expert to assign them all or part of the responsibility. The responsible group is thus comprised of those who have committed an error deemed necessary (but not sufficient) for the accident’s occurrence. With this approach, it is possible to have several responsible parties to the same accident.

The group of non-responsible parties includes drivers whose responsibility was ranked 4 or 5. These drivers are deemed fortuitously involved in the accident, through the misfortune of being in the wrong place at the wrong time.

From the concept of responsibility, this study may be considered from an epidemiological standpoint as a case-control study. The source population comprised all drivers who use public roads or private roads open to public traffic, and the two groups were drawn from this population since they had been involved in an accident that satisfied this inclusion criterion.

### Measures of association

Factors positively linked to responsibility can be interpreted as factors facilitating occurrence of the accident [24,25], on condition that they satisfy certain criteria, besides significant statistical association [26]. In practice, the significance of a risk factor for an accident (fatal or physical injuries) is gauged, as for a classic case-control study, by an odds ratio (OR) and an attributable risk (AR). The OR is a good approximation of the corresponding relative risk (RR), provided that the control group can be considered as representative of drivers. More precisely, we apply a model (logistic regression) to estimate the effect of each variable, either by itself (crude OR) or adjusted to fit the other variables retained in the model (adjusted OR). The total effect of each variable is tested for the maximum likelihood ratio at the 5% level between the model including this variable and the model which does not include it. The relative risks are estimated by the odds ratios (OR) with 95% confidence intervals. An OR is deemed significantly different to 1 if its confidence interval does not include the value 1. The calculations are made using SAS software, version 9.4 (SAS 2008).

The prevalence among the population of driving under the influence (of alcohol and cannabis) is estimated from the control population (always assuming that drivers have a very weak probability of having an accident when traveling).

The attributable risk is also estimated, as:

$$AR=(Pr(Acc)-Pr(Acc|\bar{E}))/(Pr(Acc))$$ (1)

By definition, it is the proportion of accidents that would be avoided in the (hypothetical) complete absence of considered exposure E (driving under the influence of alcohol or drugs):

Strictly speaking, the interpretation of this attributable risk depends on two hypotheses:

- Every fatal accident involves at least one responsible party. This is not absolutely the case, but the number of accidents with no responsible party is very low, and in such cases the role of driving under the influence is negligible.
- Accidents which would not have occurred in the absence of driving under the influence of alcohol or narcotics correspond with those to which the responsible party, or parties, would not have committed the driving fault that triggered the accident in the absence of alcohol or narcotics. In our view that is also an acceptable hypothesis.

In the absence of overlooked confounding variables, an AR formula [27,28] allows us to consider at the same time the approximation of RR by OR, multi-level exposure, the adjustment on confounding factors, and possible interaction between these factors:

$$AR=1-\sum_{j=1}^{J}\sum_{i=1}^{I}\rho_{ij}{OR}_{i|j}^{-1}$$ (2)

Each ρ_ij_ term represents the proportion of case subjects with exposure level i and adjustment factor level j.

## Results

An analysis was made of 2,870 accidents (out of 3,622 fatal accidents occurring in Metropolitan France in 2011) and of the corresponding 4,059 drivers tested for alcohol and narcotics, and of expert-determined responsibility. Exclusion (27.8% of drivers) was basically the result of a lack of narcotics screening. Among the included drivers, there was a slightly higher proportion of males and young people than in the total population of drivers involved in fatal accidents. Factors influencing inclusion did not significantly differ between responsible and non-responsible parties.

As indicated in the Materials and Methods section, the risk of being responsible for causing a fatal accident for a driver under compared to a driver not under the influence was estimated by comparing ORs between responsible (cases) and non-responsible drivers (controls). Table 1 shows estimated crude ORs according to detected substance.

**Table 1:** Prevalence and crude OR of responsibility linked to driving under the influence (n = 4,059 drivers involved in a fatal accident, source Voiesur 2011 data) *(ORs in brackets are non-significant)*.

		Drivers			
Blood concentrations	Number	Responsible	Not responsible	OR	95% CI
Number	4,059	2,569	1,490		
Cannabis: THC ≥ 1 ng/ml	325	10.7%	3.4%	3.45	2.84–5.82
Amphetamines ≥ 50 ng/ml	10	0.4%	0.1%	(5.22)	0.62–41.2
Cocaine ≥ 50 ng/ml	12	0.4%	0.1%	(6.40)	0.83–49.6
Opiates ≥ 20 ng/ml	43	1.3%	0.6%	2.21	1.06–4.61
Alcohol ≥ 0.5 g/l	788	29.5%	2.1%	19.7	20.1–56.3

With these crude ORs, it appears that the risk of being responsible for causing a fatal accident is much increased for drivers under the influence of alcohol (x 19.7). It is multiplied by 3.45 for those under the influence of cannabis, and by 2.2 for those under the influence of opiates. The risks associated with drivers under the influence of amphetamines or cocaine are increased but are not significantly different from 1: the corresponding confidence intervals are very wide owing to the low prevalence of these substances.

For cannabis and alcohol, it is possible to provide detailed results by measured dose (Table 2)

**Table 2:** Prevalence and crude OR of responsibility linked to driving under the influence of alcohol and cannabis per detected dose (n = 4,059 drivers involved in a fatal accident, source Voiesur 2011 data).

			Drivers			
		Number	Responsible	Not responsible	OR	95% CI
	Number	4,059	2,569	1,490		
cannabis						
	THC < 1	3.734	89.3%	96.6%	1.00	
	1 ≤ THC < 3 ng/ml	159	5.0%	2.1%	2.59	1.74–3.86
	3 ≤ THC < 5 ng/ml	64	2.3%	0.3%	7.41	2.97–18.5
	THC ≥ 5 ng/ml	102	3.4%	0.9%	3.95	2.24–6.96
alcohol						
	ALC < 0.5 g/l	3.271	70.5%	97.9%	1.00	
	0.5 ≤ ALC < 0.8 g/l	65	2.3%	0.4%	7.92	3.41–18.4
	0.8 ≤ ALC < 1.2 g/l	163	5.9%	0.8%	10.13	5.61–18.3
	1.2 ≤ ALC < 2 g/l	293	11.1%	0.5%	28.68	14.2–58.1
	ALC ≥ 2 g/l	267	10.2%	0.3%	42.15	17.4–102.3

As expected, the risk increases with the measured amount of alcohol. It is noteworthy that the much higher risks for ≥1.2g/l correspond to a higher prevalence (21.3%) among responsible drivers (71% of responsible drivers had a blood alcohol level beyond the legal limit). This dose effect was not found for cannabis, where risk was higher for the “intermediate” class.

Table 3 shows the prevalence of cannabis and alcohol according to adjustment factors to be introduced in the model.

**Table 3:** Prevalence of cannabis and alcohol among cases (responsible) and controls (not responsible) according to co-factor modalities (data source Voiesur 2011, fatal accidents).

			Cannabis prevalence (≥ 1 ng/ml)		Alcohol prevalence (≥0.5 g/l)	
		*number*	responsible	not responsible	responsible	not responsible
	Number	4,059	2,569	1,490	2,569	1,490
Sex						
	Male	3,323	12.2%	3.8%	32.7%	2.2%
	Female	736	3.0%	1.6%	13.1%	1.6%
Age*						
	≤ 24 yrs	893	15.7%	8.8%	31.0%	2.2%
	25–34 yrs	876	19.1%	7.0%	35.5%	2.3%
	35–69 yrs	2,007	5.0%	1.3%	30.0%	2.1%
	≥ 70 yrs	271	0%	0%	4.3%	0%
Vehicle category						
	Moped	149	17.1%	15.6%	47.9%	6.3%
	Motorbikes	557	13.9%	8.9%	32.6%	4.8%
	Light vehicle	2,367	10.9%	3.5%	31.7%	2.7%
	Van	313	3.8%	1.3%	17.6%	0%
	HGV	471	5.8%	1.2%	1.5%	0%
	Other	202	4.6%	2.1%	16.7%	3.2%
Day and time						
	Weekday	2,339	7.4%	2.6%	14.6%	1.1%
	Saturday	448	8.5%	4.5%	28.9%	1.9%
	Sunday	403	7.0%	3.0%	34.4%	1.5%
	weekday nights	451	19.6%	4.1%	48.4%	4.8%
	Friday/Saturday nights	209	22.0%	12.0%	65.4%	8.0%
	Saturday/Sunday nights	209	21.0%	4.3%	77.8%	8.5%

*Age missing for 12 drivers thereafter excluded from analysis

The prevalences of cannabis and alcohol appear lower for women. The prevalence of cannabis was clearly lower for those aged 35 and above, which was not observed for alcohol. As far as the user categories are concerned, moped riders had the strongest prevalence of cannabis and alcohol, followed by motorcyclists. Finally, prevalence was clearly greater at night, and greater still at night during weekends.

Table 4 details the ORs for factors of interest according to measured dose, adjusted for the four preceding factors.

**Table 4:** Adjusted ORs* for driver responsibility linked to driving under the influence (n = 4,047**, data source Voiesur 2011, fatal accidents).

	OR	95% CI
THC < 1 ng/l	1	
1 ≤ THC < 3 ng/l	1.35	0.86–2.14
3 ≤ THC < 5 ng/l	3.59	1.36–9.48
THC ≥ 5 ng/l	1.59	0.85–2.97
All doses THC ≥ 1 ng/ml	1.65	1.16–2.34
ALC < 0.5 g/l	1	
0.5 ≤ Alc <0.8 g/l	6.40	2.70–15.2
0.8 ≤ Alc < 1.2 g/l	8.30	4.52–15.2
1.2 ≤ Alc < 2 g/l	24.4	11.9–50.1
Alc ≥ 2 g/l	44.4	18.1–109
All doses ALC ≥ 0.5 g/l	17.8	12.1–26.1
OPI < 20 ng/ml	1	
OPI ≥ 20 ng/ml	2.21	1.02–4.78

* Adjustment factors included in the model: age, gender, vehicle category, time of accident

** Known alcohol and narcotic status: responsibility determined by expert and age known (2,562 cases and 1,485 controls)

The effects of cannabis, alcohol and opiates remained significant after adjusting for all of the co-factors likely to have an influence on responsibility. All first-order 1 interactions were tested. None were significant. In particular, we found no significant interaction of alcohol x cannabis (p = 0.29).

Table 5 shows estimated attributable risks calculated from the above adjusted estimates.

**Table 5:** Risk risks attributable* to driving under the influence (n = 4,047**. data source Voiesur 2011, fatal accidents).

	Attributable risk	95% CI
THC ≥ 1 ng/ml	4.20%	3.71%–4.75%
ALC ≥ 0.5 g/l	27.7%	26.0% -29.4%
OPI ≥ 20 ng/ml	0.73%	0.49%–0.98%

* Adjustment factors included in the model: age, gender, vehicle category, time of accident

** Known alcohol and narcotic status: expert-determined responsibility and age known (2,562 cases and 1,485 controls)

The risk fraction attributable to driving under the influence of alcohol appears very high, 27.7%, while it is estimated at only 4.2% for driving under the influence of cannabis, and for all doses. (The fraction for opiates is lower still, 0.73%.).

As indicated above, there was no significant alcohol x cannabis interaction. This means that the increased risk of being responsible for a fatal accident due to alcohol does not differ significantly whether the driver is or is not also under the influence of cannabis (and vice versa). In turn, that means the odds ratio for a driver under the influence of both alcohol and cannabis can be estimated by the product of the OR relative to alcohol and the OR relative to cannabis. Table 6 distinguishes between drivers under the influence of alcohol alone, cannabis alone, alcohol and cannabis or neither.

**Table 6:** Number and prevalence for isolated or combined influences for responsible and non-responsible drivers (n = 4,047*, data source Voiesur 2011).

	N	Not responsible prevalence	Responsible prevalence
Neither THC nor alcohol (THC < 1 ng/ml & ALC < 0.5g/L)	3,096	94.9%	65.8%
THC alone (THC ≥ 1 ng/ml & ALC < 0.5g/L)	166	3.0%	4.8%
Alcohol alone (THC < 1 ng/ml & ALC ≥ 0.5 g/l)	627	1.8%	23.5%
THC and alcohol (THC ≥ 1 ng/ml & ALC ≥ 0.5 g/l)	158	0.3%	6.0%

* Known alcohol and narcotic status: expert-determined responsibility and age known (2,562 cases and 1,485 controls)

Thus, more than half of the responsible drivers under the influence of cannabis were also under the influence of alcohol.

As influence of cannabis was often associated with influence of alcohol, we compared separately "THC only" drivers with the reference category "no substance" (no alcohol, no cannabis and no other drug), and "Alcohol only" drivers with the same reference category. As expected because no multiplicative interaction between alcohol and cannabis was found, separate estimates were very close to those of the global analysis shown Table 4, using the same adjustment factors, i.e. OR = 1.72 (95% CI:1.18–2.51) for THC all doses and OR = 19.7 (95% CI: 12.9–30.1). Concerning specifically the "alcohol free" analysis, OR associated with detected dose were also close to the previous ones (1.52 for 1 ≤ THC < 3 ng/l, 3.23 for 3 ≤ THC < 5 ng/l, 1.55 for THC ≥ 5 ng/l), which means that the middle dose of cannabis being associated with the highest risk was not due to a higher alcohol dose in the middle group of drivers under the influence of cannabis.

## Discussion

In common with most published studies, we found that a driver under the influence of alcohol has a much higher risk of contributing to a fatal accident. The marked dose-effect suggests a causal role, perfectly consistent with the demonstrated effects of alcohol intoxication, which are essentially a weakening of the capacities necessary for safe driving and an increase in self-confidence that pushes the driver to over-estimate his or her capacities, in particular, for driving at higher or unsuitable speeds [29]. Noteworthy, too, is that more than two thirds of responsible drivers were well above the legal limit (≥1.2g/l) and one third were even above 2g/l. The risks associated with these levels of alcohol intoxication are very high, and this explains, according to the AR formula, why the fraction attributable to driving under the influence of alcohol was close to 28%. This result accords with several published studies which estimate that driving under the influence of alcohol is to blame for one third of all road deaths [29].

Very comparable results were found in a similar study (SAM) of fatal accident data from October 2001 to September 2003 in France, of which the principal results were published in the BMJ [16]. The fraction attributable to alcohol was estimated at 31.5% (95% CI: 30.7%-32.3%), with the same dose-effect and a higher prevalence of strong intoxication among responsible drivers. Only the risk for all doses taken together was lower (8.51; 95% CI: 7.15–10.1). However, the methodology of the SAM study was somewhat different, and the estimates of the present study were recalculated applying the methods of the SAM study in order to assess the effect of these methodological differences on the results, as reported in S1 Appendix. They make clear that only the method of determining responsibility had a significant influence on the results. If one applies the previous method to the current study, differences in risk estimation become negligible.

As regards driving under the influence of cannabis, the attributable risk fraction was considerably lower than for alcohol, but significant for all doses taken together, with no apparent dose-effect. Here again, the attributable risk fraction (4.2%; 95% CI: 3.7–4.8) was close to that of the previous study (4.3%; 95% CI: 2.9–5.8). The estimates of prevalence and risks were also close: respectively, 3.4% (95% CI: 2.9–3.9) vs. 2.8% (95% CI: 2.2–3.4), and 1.65 (95% CI: 1.16–2.34) vs 1.78 (95% CI: 1.40–2.25). In the previous study, a slight dose-effect emerged. The fact that the results of two studies are inconsistent is not totally surprising. Unlike alcohol, the level of intoxication from cannabis at the time of the accident is quite difficult to estimate, owing to the strong increase in THC concentration just after consumption followed by a rapid decrease.

OR estimates in the recently published meta-analyses cited in the Introduction are of the same order of magnitude as those of the present study, although comparison is made difficult by differences in target populations and sampling, and sometimes by wide divergences in study design, methods of screening (and of confirmation of screening results), and of taking account of confounding factors (5, 9–11).

In their recent paper [30], Rocheberg and Elvik used an original measure of association. Compared to the standard OR, they used the same denominator (intoxicated / non intoxicated among non responsible drivers), but they used the numerator we would obtain by comparing drivers involved in a crash to drivers not involved in a crash (intoxicated drivers among drivers involved in a crash / non intoxicated drivers involved in a crash whatever they are responsible or not). Their objective was to improve the comparability of the estimations coming from the two main study designs (case-control studies where controls are not involved in a crash and responsibility studies). In the present study, adding non responsible drivers to the numerator (which is generally done in case-control studies because responsibility is not assessed) could result in deflecting from the objective of finding causal factors. In addition, this new estimator raises concerns for variance estimation. Furthermore, we used ORs to estimate attributable risks, which is only valid for causal factors.

Among these confounding factors, it is essential that alcohol be taken into account since very often (more than one in two times, according to our data) the consumption of cannabis is accompanied by consumption of alcohol. Put another way, a study that does not consider alcohol has every chance of overestimating the effect of cannabis, since the effect of cannabis on driving is going to be, for around half of drivers, the reflection of alcohol consumption, which itself is linked to a strongly heightened risk of accident responsibility.

More generally, while the effect of consuming alcohol on driving has been well established by both experimental and observational studies, the effect of consuming cannabis is more controversial. There are several reasons for the volatility of results relating to cannabis. The effects of cannabis on individuals vary more than those of alcohol, as a result of differences in individual tolerance, techniques of consumption, and concentrations [31]. Furthermore, proof of cannabis consumption is harder to obtain, and its effect on driving is without doubt less strong than for alcohol.

As regards research on other psychotropic substances (besides alcohol), results confirm the rarity of exposure to amphetamine and cocaine, and the impossibility of determining the associated risks from our data. On the other hand, and contrary to the previous study, driving under the influence of opiates appears linked to a significant risk, with an adjusted OR of 2.2, prevalence of 0.6% and attributable risk estimated at 0.7%. However, the small number of drivers under the influence of opiates calls for caution in interpreting these results, especially as it cannot be completely ruled out that, in some cases, the presence of opiates may be due to a post-trauma injection of morphine.

### Study limitations

The value of the present results depends to a large extent on the study design: the quality of determining responsibility, of selecting the control group.

We were able to confirm that the instructions given to the experts not to take into account the fact that certain drivers were under the influence (tested positive to alcohol or to drug screening) were respected. Likewise, agreement between experts in attributing responsibility was confirmed. Details of these two points are developed in S2 Appendix.

Concerning the composition of the control group, the analysis of responsibility was based on the hypothesis that drivers considered not responsible for an accident make up a random sample of the general population of drivers [32]. If this is valid, interpreting the ORs should show the increased risk of being responsible for a fatal accident as compared to all active drivers, rather than to drivers involved in a fatal accident.

Strictly speaking, this hypothesis is impossible to confirm, since it would require data on those not involved in an accident but with the same exposure characteristics. In the absence of such a group, the selected control group comprised all non-responsible drivers involved in fatal accidents. The advantage of this lies in having the same quality of information as for responsible drivers and, for accidents involving two or more vehicles, homogeneity for certain circumstances of the accident (same place, same time, same traffic conditions).

However, having a control group comprising drivers involved in an accident for which they were not responsible implies selecting control subjects according to the severity of the accident (fatal in this case), although this severe outcome was to a large extent caused by the speed of the vehicles(s) at the time of collision. Yet various factors of interest are also linked to this speed factor, such as driving under the influence (of alcohol in particular), or the gender or age of the driver. Adjusting for some of these factors reduces but does not completely eliminate bias [33].

According to the French Monitoring Center for Drugs and Drug Addiction (OFDT) [34], the proportion of cannabis users remained stable between 2000 and 2014. In hypothesizing that the proportion of users driving while having recently consumed cannabis also did not vary appreciably, the prevalence of drivers under the influence can be expected not to vary greatly. That is what we observed, with a prevalence estimated at 3.4%, close to that observed in the SAM study (2.8%), although no other figure is available in the literature to support this result. It was from this same group that alcohol prevalence was estimated, at 2.1%, which is quite close to the prevalence observed in the SAM study (2.7%), and found in Belgium (2.7%) in a large survey of drivers [35]. Consumption patterns no doubt differ between Belgium and France, but the similarity of the estimated prevalence is a step in the right direction as far as the appropriateness of using the non-responsible group to approximate the general active driver population is concerned.

Furthermore, excluding certain drivers in whom narcotics were not assessed may have distorted the estimation of prevalence and ORs. This exclusion most often concerned elderly and female drivers, and could lead to a slight overestimation of prevalence since it was individuals liable to show lower prevalence who were excluded (Table 3). This is not a problem for the ORs, thanks to adjustment for factors including age and gender.

Another concern is that THC and alcohol blood concentrations decrease rapidly after smoking or drinking (12,21). Time intervals between the crash, the screening and the blood collection were not available in our data. While the time lag between the crash and the screening can reasonably be supposed to be short, blood collection can occur quite a long time after the crash, which can shift a number of THC or alcohol cases below the threshold. The consequence on OR estimates must be limited because this time lag can be considered as independent of the responsibility, but it can lead to an underestimation of THC and Alcohol prevalences and then of attributable fractions.

One last limitation can be mentioned. No information on the taking of medication, and in particular medication with a known or possible effect on driving [36–38], was available.

## Conclusion

Besides numerous developments during the almost ten years that separate the present study and the one carried out in 2001–2003, the prevalence of drivers responsible for causing fatal accidents while under the influence of alcohol or narcotics has remained remarkably stable, and with it the proportion of fatal accidents which could in theory have been avoided if no drivers ever exceeded the legal limits. Road deaths strongly declined over this period, mainly thanks to lower speeds [39,40], and the number of victims attributable to alcohol and/or cannabis has diminished by the same proportion as for all road deaths. This result suggests that drivers under the influence have reduced their speeds in the same way as those who are sober.

Alcohol remains the main problem in France. It is just as important to note that one in two drivers considered to be under the influence of cannabis was also under the influence of alcohol (while 20% under the influence of alcohol were also under the influence of cannabis). With risks cumulating between the two, the message of the particular danger of conjointly consuming alcohol and narcotics (particularly cannabis) is as relevant as ever.

## Supporting information

S1 Appendix (DOCX)

S2 Appendix (DOCX)
