# Supplementary material for: Cannabis, alcohol and fatal road accidents
Source: PLoS One. 2017 Nov 8;12(11):e0187320. doi: 10.1371/journal.pone.0187320 (PMC5678710; doi:10.1371/journal.pone.0187320)
Supplement: S1 Appendix — (DOCX) [file pone.0187320.s001.docx]

## Appendix S1: Assessment in the present study of the effect of methodological choices made in a similar study carried out in 2001-2003

The two studies differ on three methodological points. The impact of these choices on the results obtained is as follows:

The first point is the choice of reference level, which included persons having positive blood concentrations but below the legal limit (mainly deceased persons with systematic blood test). This choice has a negligible influence on the results, because the number of these persons is very low compared with the number of persons who tested negative.

The second consists in the exclusion of non-responsible deceased persons who were the only fatalities in the accident. This exclusion was motivated by the fact that the probability of being killed was higher in drivers who tested positive for alcohol and/or cannabis. Thus, non-responsible drivers were more likely to be included in the control group, because their risk of being killed, and therefore of being involved in a fatal accident, was higher. As this phenomenon was much less frequent in the present 2011 data, and the statistical power of the present study was further reduced by the fact that it was limited to a single year’s accidentology data, with a lower annual number of fatal accidents than 10 years previously, the present control group comprised all non-responsible drivers involved in a fatal accident. Applied to the present study, this reduction in the non-responsible group led to an increase in the ORs related to alcohol and cannabis, but which remained in the same order of magnitude.

Thirdly, the basic difference stems from a less specific determination of responsibility, because expert advice was obtained for only a small part of the observations. In order to assess what influence this difference made to the results, the following Tables replicate Tables 2 and 4, using responsibility based on Robertson and Drummer’s method (R&D), as in the 2002-2003 SAM data set, in place of responsibility judged by experts.

S1 Table 2-A: Prevalence and crude OR of responsibility (R&D) linked to driving under the influence
(n=3,922 drivers involved in a fatal accident. Voiesur 2011 data)

|  |  | Drivers | |  |  |
| --- | --- | --- | --- | --- | --- |
| Blood concentrations | Number | Responsible  (R&D) | Non-responsible  (R&D) | OR | 95% CI |
| Number | 3,922 | 2,976 | 1,246 |  |  |
| Cannabis. THC ≥ 1 ng/ml | 317 | 10.3% | 3.4% | 3.28 | 2.36 – 4.58 |
| Amphetamines ≥ 50 ng/ml | 10 | 0.3% | 0.2% | (1.86) | 0.40 – 8.77 |
| Cocaine ≥ 50 ng/ml | 12 | 0.4% | 0.2% | (2.33) | 0.51 – 10.7 |
| Opiates ≥ 20 ng/ml | 43 | 1.2% | 0.8% | (1.54) | 0.76 – 3.14 |
| Alcohol ≥ 0.5 g/l | 775 | 27.0% | 4.2% | 8.50 | 6.36 – 11.4 |

Compared to the present Table 2, the OR related to opiates is no longer significant, that related to cannabis is similar, and that related to alcohol is weaker.

S1 Table 4-A: Increased risk of driver responsibility when driving under the influence (legal limits): OR adjusted for all co-factors^*^ and prevalences
(n=3,910^**^. Voiesur 2011 data, fatal accidents)

|  | OR | 95% CI |
| --- | --- | --- |
| THC < 1 ng/l | 1 |  |
| 1 ≤ THC < 3 ng/l | 1.40 | 0.87 -2.23 |
| 3 ≤ THC < 5 ng/l | 1.92 | 0.84 -4.42 |
| THC ≥ 5 ng/l | 2.47 | 1.20 -5.09 |
| All doses THC ≥ 1 ng/ml | 1.72 | 1.20 -2.48 |
| Alc < 0.5 g/l | 1 |  |
| 0.5 ≤ Alc <0.8 g/l | 5.60 | 2.22 -14.1 |
| 0.8 ≤ Alc < 1.2 g/l | 6.52 | 3.48 -12.2 |
| 1.2 ≤ Alc < 2 g/l | 6.06 | 3.80 -9.66 |
| Alc ≥ 2 g/l | 10.4 | 5.93 -18.1 |
| All doses ALC ≥ 0.5 g/l | 7.27 | 5.33 -9.93 |
| OPI < 20 ng/ml | 1 |  |
| OPI ≥ 20 ng/ml | 1.48 | 0.71 -3.09 |

^*^Co-factors included in the model: age, gender, vehicle category, time of accident
^**^Known alcohol and narcotic status, R&D responsibility determined and age known (2,670 responsible, 1,240 non-responsible)

Concerning the adjusted OR values, two differences are to be noted: the effect related to opiates is no longer significant, and the effect related to alcohol is still high, but much less than with the use of expert-assessed responsibility. Even so, as explained in Appendix S2, we were able to confirm that the experts who determined responsibility did not take account of drivers’ alcohol status (which, of course, would have led to an over-estimation of the alcohol risk). It is therefore likely that the use of responsibility in Roberston and Drummer’s sense leads to an under-estimation of the OR for driving under the influence of alcohol, taking much less account of the driving errors often made by drivers under the influence of alcohol.
