# Supplementary material for: Cannabis, alcohol and fatal road accidents
Source: PLoS One. 2017 Nov 8;12(11):e0187320. doi: 10.1371/journal.pone.0187320 (PMC5678710; doi:10.1371/journal.pone.0187320)
Supplement: S2 Appendix — (DOCX) [file pone.0187320.s002.docx]

## Appendix S2: Validation responsibility determination

Concerning the effective quality of responsibility determination by experts, two key points were examined:

- The respect accorded to the instruction given to the experts not to take into account the fact that certain drivers were under the influence (positive alcohol test or drug screening). For that, responsibility was statistically modeled from a learning sample comprising accidents involving two vehicles, for which the two drivers tested negative for alcohol and the expert-determined responsibility was known (n = 3,878 drivers). This model was then applied to a test sample composed of other accidents involving two vehicles (n = 2,648 drivers). Overall, the numbers of responsible drivers predicted by the model (E) and observed by the expert (O) were close (ratio E/O = 1.00). Furthermore, these predicted and observed numbers remained close for drivers who were under the influence of alcohol (ratio E/O = 0.93) and those who were not (ratio E/O = 1.19). This result suggests that experts had not (or only to a negligible extent) directly taken account of drivers’ alcohol status.

- *Inter-expert agreement in attributing responsibility:* A special method [1] was required to deal with the fact that each case of responsibility was determined by only 1 of the 18 experts. The principle of this method is as follows: a “sparse” logistic regression was used to find out the responsible/non- responsible classification rule used by each expert from 205 available variables (describing the characteristics of the driver and the circumstances of the accident). More precisely, for the *k*^th^ expert, we estimated the vector of parameters $\beta_{k}$ under the model $logit P\left( Resp=1 \right|X, Z=k)= \beta_{k}^{T}X,$ where *Resp* is the binary variable indicating that the driver was judged responsible by an expert, *X* designates the vector of 205 available variables (as well as the constant 1, for the model intercept) and *Z=k* indicates that the accidents of interest are exclusively those for which responsibility was determined by the *k*^th^ expert. The approach developed in [2] enables simultaneous estimation of the 18 vectors $\beta_{1}, \ldots, \beta_{18}$, taking advantage of the expected homogeneity between vectors while identifying any heterogeneities. In the present case, no heterogeneity was identified, suggesting that the various experts used similar classification rules to determine the responsibility of drivers.

# References

1. Ollier, E & Viallon, V 2014. ‘Joint estimation of K-related regression models with simple L_1 -norm penalties,’ *arXiv preprint arXiv: 1411.1594*

2. Ollier, E & Viallon, V 2017. ‘Regression modelling on stratified data with the lasso’, Biometrika, Vol 104 (1) pp 83–96
